# Development of Triamcinolone Acetonide-Loaded Nanostructured Lipid Carriers (NLCs) for Buccal Drug Delivery Using the Box-Behnken Design

**DOI:** 10.3390/molecules23040982

**Published:** 2018-04-23

**Authors:** Pakorn Kraisit, Narong Sarisuta

**Affiliations:** Division of Pharmaceutical Sciences, Faculty of Pharmacy, Thammasat University, Pathumthani 12120, Thailand; narong_s@tu.ac.th

**Keywords:** buccal drug delivery, Box-Behnken design, triamcinolone acetonide, nanostructured lipid carriers (NLCs), confocal laser scanning microscopy (CLSM), Nile red

## Abstract

The aim of this present work was to prepare triamcinolone acetonide (TA)-loaded nanostructured lipid carriers (TA-loaded NLCs) for buccal drug delivery systems using the Box-Behnken design. A hot homogenization method was used to prepare the TA-loaded NLCs. Spermaceti (X_1_), soybean oil (X_2_), and Tween 80 (X_3_) were used as solid lipid, liquid lipid, and stabilizer, respectively. The particle size of TA-loaded NLCs was lower than 200 nm and the zeta potential displayed the negative charge in all formulations. The percentage encapsulation efficiency (%EE) of the TA-loaded NLCs showed that it was higher than 80% for all formulations. Field emission scanning electron microscope (FESEM) confirmed that the size of TA-loaded NLCs was approximately 100 nm and energy-dispersive X-ray spectroscopy (EDS) confirmed that the TA could be incorporated in the NLC system. The Higuchi model gave the highest value of the R^2^, indicating that this model was a fit for the TA release profiles of TA-loaded NLCs. Confocal laser scanning microscopy (CLSM) was used to observe the drug penetration within the porcine buccal mucosa and Nile red-loaded NLCs showed significantly higher penetration depth at 8 h than at 2 h. Therefore, TA-loaded NLCs could be an efficient carrier for drug delivery through the buccal mucosa.

## 1. Introduction

Over the last decades to the present day, the buccal mucosa route has been extensively investigated for the delivery of various drugs, peptides, and macromolecules for local or systemic treatment because of its many advantages, such as the avoidance of drug degradation in the GI tract, avoiding the first pass metabolism, and the high blood supply [1,2]. Additionally, non-keratinized parts of the human buccal mucosa show a higher rate of absorption when compared with the skin and other keratinized epithelium [3,4,5]. However, an important limitation of the drug delivery through the buccal mucosa is the low permeation leading to low bioavailability [1,2]. To increase the drug permeability, the drug delivery using a nanoparticle is an interesting approach to overcoming the limitation.

Presently, nanostructured lipid carriers (NLCs) are a popular and well-researched nanoparticle preparation used in many fields of pharmaceutics, including many routes for drug delivery, such as topical skin, ocular, pulmonary, parenteral injection, and oral administration including buccal mucosa [3,6,7,8]. NLCs are the second generation of lipid nanoparticles composed of a mixture of solid and liquid lipids [9,10,11]. NLCs have displayed many advantages over conventional carriers, such as increased permeability and bioavailability, reduced adverse effect, and large-scale production. Further, they avoid using organic solvents and increase drug solubility and drug targeting [3,10,12,13]. Additionally, the high efficiency of NLCs to encapsulate hydrophobic drugs is a considerable advantage over the lipid first generation (solid lipid nanoparticles, SLNs) due to its less ordered crystalline structure of lipid matrix [3,14,15]. This structure of NLCs also avoids faster drug release and drug expulsion during the storage compared with SLNs [16,17]. Therefore, NLCs probably increases the penetration across the buccal mucosa and high entrapment efficiency of hydrophobic drugs, such as triamcinolone acetonide (TA).

**Figure 1:**
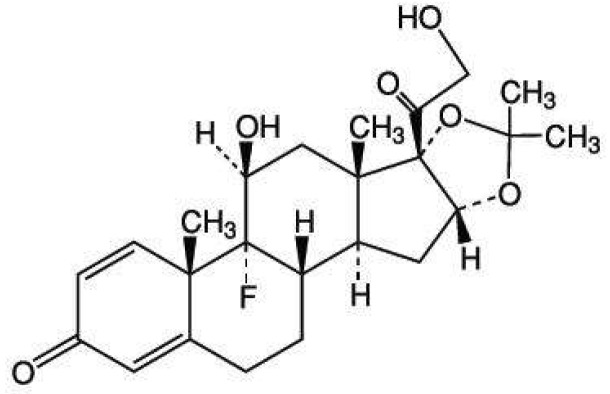
The structure of triamcinolone acetonide.

TA (Figure 1) is a synthetic glucocorticoid drug commonly used to treat many diseases, such as arthritis, oral inflammation, and allergies [18,19]. Several methods are used. A traditional method involves the trial and error methodology to prepare TA formulations [4,11,20]. However, this approach is quite time-consuming, expensive, laborious, and unpredictable [21,22]. Response surface methodology (RSM) is used to solve these problems and it can be optimized in the formulation of many drug deliveries [21,22,23]. RSM’s main benefit is concurrently analyzing the variables when factor interactions are complex. The Box-Behnken design is a type of RSM most commonly used to optimize the formulations [23,24] because when compared with other designs, such as central composite, it requires fewer runs and less time when three independent variables are examined [23,24].

The objective of this present work was to prepare TA-loaded NLCs for buccal drug delivery system using Box-Behnken design. Soybean oil and spermaceti were selected for use in this study as liquid and solid lipid, respectively, because of their low cost, high abundance, biocompatibility [14]; and show high miscibility in concentrations using NLCs formation (data not shown). To stabilize NLCs, Tween 80 was used as a non-ionic stabilizer because of its widespread use in the pharmaceutical field, low toxicity, less irritation to the cell membrane, and ability to increase permeability [3,25]. Moreover, Tween 80 is more stable for NLC dispersion than other stabilizers [3]. A hot homogenization method was used to prepare the NLCs and characterize it in terms of particle size, zeta potential, in vitro release study, and entrapment efficiency (%EE). A field emission scanning electron microscope (FESEM) and energy-dispersive X-ray spectroscopy (EDS) were used to investigate the morphological characteristic and identified TA of selected NLCs. Permeation studies using porcine buccal mucosa were performed and confirmed by CLSM imaging.

## 2. Results and Discussion

### 2.1. Physicochemical Characterization of TA Loaded NLC (Zeta Potential and Particle Size)

**Figure 2:**
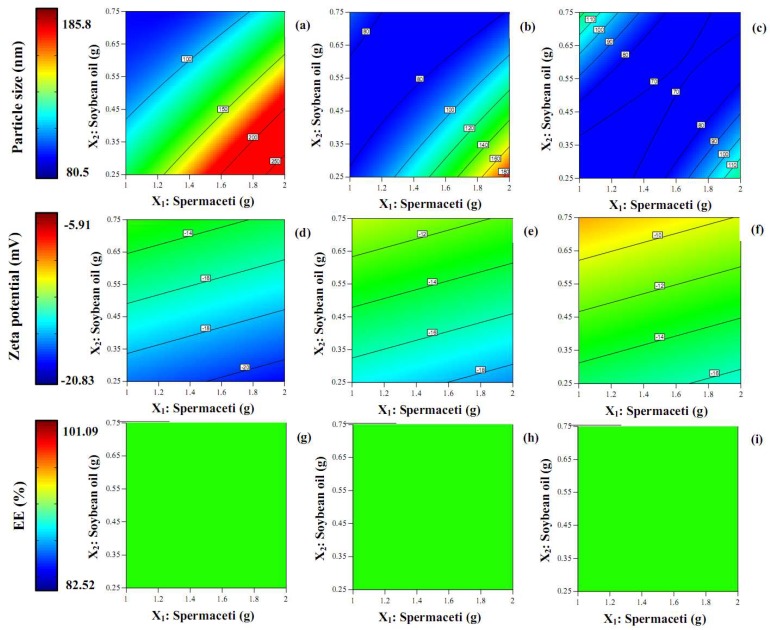
The contour plots of the triamcinolone acetonide-loaded nanostructured lipid carriers for particle size (**a**–**c**); zeta potential (**d**–**f**); %EE (**g**–**i**) and Tween 2 g (left column), Tween 3 g (middle column), and Tween 4 g (right column).

Generally, in recent decades to the present-day, nanoparticle research in the pharmaceutical field focused on two physical important factors that must be examined: particle size and zeta potential. In this study, in Table 1, the particle size of TA-loaded NLCs was lower than 200 nm and is displayed in the size range 80.5–185.8 nm. Figure 2 shows the contour plot for the effect of the concentration of spermaceti, soybean oil, and Tween 80 on the particle size. The increase in the spermaceti concentration and concentration of Tween 80 was fixed, leading to a great increase in the particle size. In contrast, with an additional amount of soybean oil and a fixed concentration of Tween 80, the particle size was reduced. The result was associated with another report that increased the amount of liquid lipid and saw the size of the particle decrease. These appearances may be due to the reduction in the viscosity of the internal phase [9]. Nevertheless, other factors may affect this appearance, such as the chemical interaction of components or the molecular structure of the lipids used [12]. The larger blue area in the contour plot of Figure 2c than in Figure 2a,b shows that the particle size was smaller when the concentration of the Tween 80 that increased. The result correlated with the result of increasing the amount of liquid lipid: the particle size was smaller when it was higher. The increase in the amount of non-ionic surfactant that provided the TA-loaded NLCs with a smaller size may be due to the decrease in the interfacial tension between the oil and aqueous phases, resulting in the smaller size of droplet formation [9,14].

Table 1 shows the zeta potential of all TA-loaded NLC formulations, which ranged between −5.91 to −20.83 mV and displayed the negative charge in all preparations because of their carboxylic molecules from the lipid structure [3,12]. The contour plot for the effect of the concentration of spermaceti, soybean oil, and Tween 80 on the zeta potential is in Figure 2d,f. The reduction of the zeta potential (higher negative charge) could be observed at the higher amount of solid lipid, but it was insignificant (*p* > 0.05). Conversely, the zeta potential was higher with the increase of liquid lipid incorporating. The blue area of the contour plot in Figure 2d was more than in Figure 2e,f, indicating that the zeta potential increased when a higher amount of Tween 80 was loaded in the TA-loaded NLCs (*p* > 0.05).

**Figure 3:**
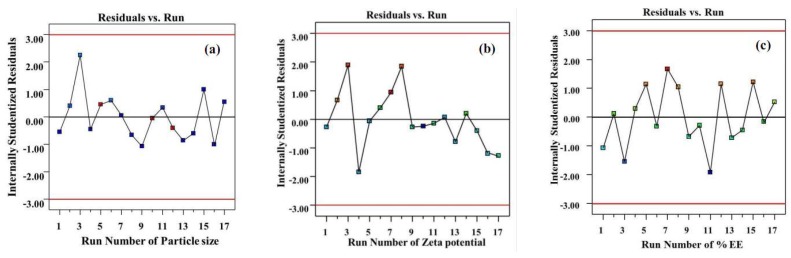
The corresponding residual plot between the run number and the internally studentized residuals for various responses of particle size (**a**); zeta potential (**b**); and % EE (**c**).

The corresponding residual plot between the run number and the internally studentized residuals of Y_1_ and Y_2_ are shown in Figure 3a,b, respectively. This was employed to test the reliability of the dependent variables. In the totally randomized run, the vertical spread of the internally Studentized residuals was in line from bottom-to-top, implying that all the data points lay within the limits of a 95% confidence interval [21].

### 2.2. Determination of Entrapment Efficiency

Table 1 displays the percentage of encapsulation efficiency (%EE) of TA-loaded NLCs, which were in the range of 82.52–101.09%, indicating that the NLC system had a highly effective encapsulation of TA. The high loading ability of the NLCs may be due to the hydrophobicity of TA, including the interaction between the TA and the lipid components [14]. See Figure 2g–i for the contour plots of the effect of the concentration of spermaceti, soybean oil, and Tween 80 on the %EE. The difference in the concentration of the three components showed the only green area in the contour plot. It could be concluded that the %EE did not significantly (*p* > 0.05) depend on the concentrations of the lipids and the surfactant in the NLC system. This result related to other previous reports that the encapsulation efficiency of drugs loaded in the NLCs matrices is over 80% and the amount of each substance does not significantly influence the %EE [3,14,26]. In Figure 3c, the reliability of the Y_3_ was analyzed by comparing the run number versus the internally studentized residuals and displaying the data points that lay within the limits.

### 2.3. Field Emission Scanning Electron Microscope (FESEM) and Energy-Dispersive X-ray Spectroscopy (EDS)

**Figure 4:**
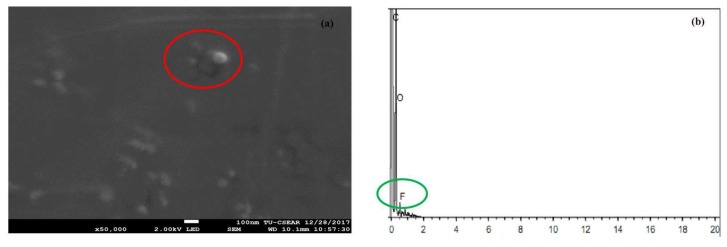
The field emission scanning electron microscope image (**a**) and energy-dispersive x-ray spectroscopy spectrum (**b**) of the TA-loaded NLCs.

FESEM was used to investigate the morphology and size of the particles from TA-loaded NLCS (see Figure 4). The N2 formulation was selected to be examined in this study and showed quite a spherical shape in the nanosize range. Each particle size of the nanoparticles was around 100 nm and correlated with the result of the measurement using the light scattering technique as explained in the previous section. The particles in the red circle area (only particles) of Figure 4a were detected using EDS to confirm TA could be incorporated in the NLC system. The result in Figure 4b shows the characteristic peak of fluoride (F, in green circle) of the TA structure. From the composition of the NLCs formulation, only the active ingredient displays fluoride elements in the molecular structure, see Figure 1. The TA might be loaded in NLC or attached outside the layer of NLCs. Therefore, the result could suggest that the TA could be loaded in the NLC system and it could form the nanoparticle.

### 2.4. In Vitro Release of TA Loaded NLC

**Figure 5:**
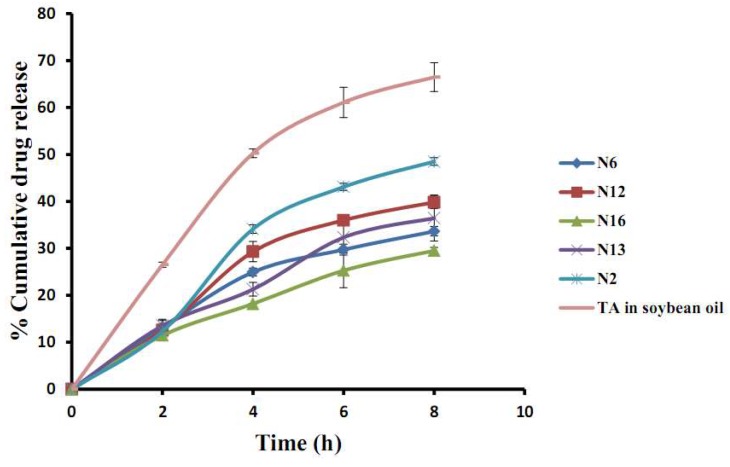
The in vitro release of TA from the TA-loaded NLCs and TA in soybean oil in simulated saliva fluid pH 6.8.

The in vitro release of TA from the NLC dispersion in the period of 0–8 h varied from 0% to 66.5%, see Figure 5. The formulations of N2, N6, N12, N13, N16, and TA in soybean oil were selected in this study to compare the effect of concentration of spermaceti (N2 and N16), soybean oil (N6 and N12), Tween 80 (N12 and N13), and TA-loaded NLCs selected in this section with TA in soybean oil. In the early release period at 2 h, the release of TA from the NLC dispersion of all formulations did not differ significantly (*p* > 0.05), but the formulation of TA in soybean oil showed a great release of TA in that period of time and it released considerably more of all the TA-loaded NLC formulations until 8 h. The result suggested that TA could be incorporated in the NLC system because they were lower release when compared with TA in soybean oil. The mechanism of the NLCs release involved two different states; first, the immediate release of nanoparticles from the surface of the NLCs and next, the diffusion through the NLCs which was the predominant release mechanism [27]. TA-loaded NLCs of N12 with a higher amount of soybean oil than N6 had a higher cumulative release after 2 h. Similar findings were present in other reports [12,26]. The result could be explained by the cooling process of the NLCs preparation after the ultrasonic method. In the period of the cool down process of NLC dispersion, the high melting point of solid lipid (spermaceti) rapidly changed to form a solid lipid matrix while the liquid lipid (soybean oil) became embedded in a solid lipid matrix or distributed around the surface of the matrix. The matrix of the particle is composed of imperfections, giving space to contain the drug in amorphous sections [26,28]. The result of the higher amount solid lipid supported this appearance: the higher amount of solid lipid in formulation N16 showed the lower cumulative release than the formulation contained in the lower amount of solid lipid in formulation N2. It could be concluded that the concentration of solid and lipid to form TA-loaded NLCs had a crucial influence on the release profile of the drug. The increase in the amount of surfactant in formulation N12 than N13 showed a higher release profile after 2 h of this experiment. It could be described by the addition of the surfactant leading to improve dispersity and solubility of a hydrophobic drug providing a higher release rate in the SSF medium. Table 2 shows the release kinetics model of the TA from selected NLC formulations. The suitable mathematical release models chosen for the release pattern were zero-order, first-order, and Higuchi models. A maximum of the correlation coefficient (R^2^) was used to select a model appropriate for analyzing the TA release patterns. The Higuchi model gave the highest value of the R^2^, indicating that this model was a fit for the TA release profiles and correlated with another report of NLCs formulation [9]. So, the release patterns of the TA-loaded NLCs fit with the Higuchi model suggests drug release by diffusion [29].

### 2.5. In Vitro Permeation Studies

**Figure 6:**
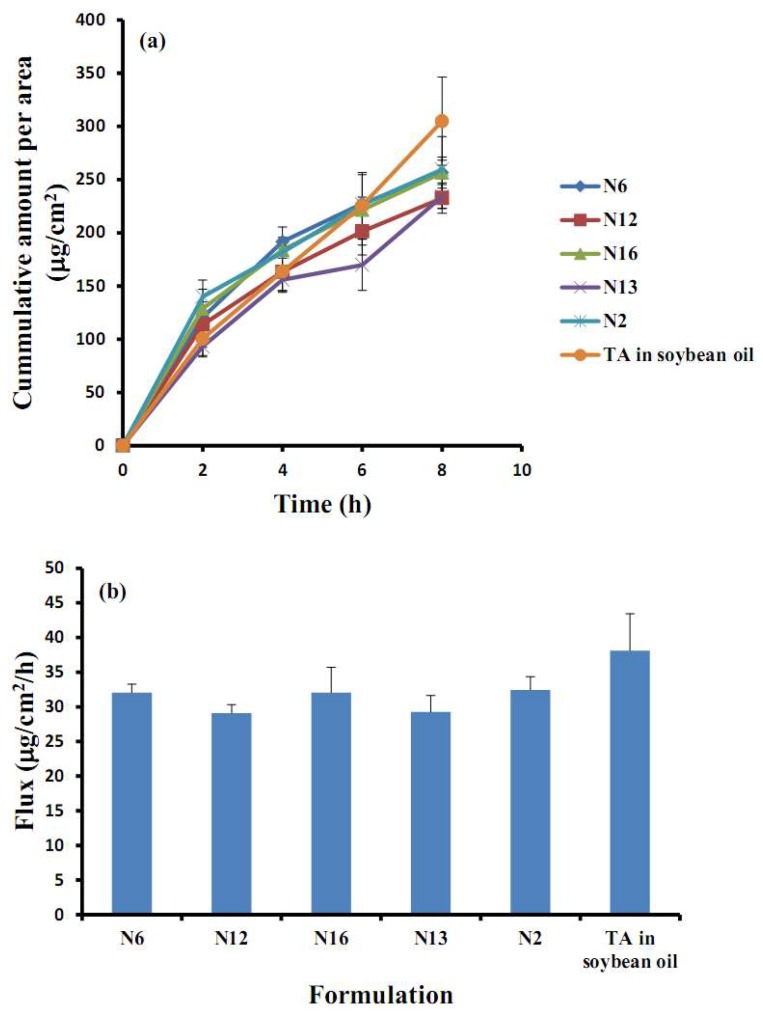
The in vitro permeation profiles (cumulative amount per area (**a**) and steady-state flux (**b**)) of TA-loaded NLCs and TA in soybean oil.

The permeation study of selected TA-loaded NLC formulations (N2, N6, N12, N13, N16) and TA in soybean oil through porcine buccal mucosa with respect to the cumulative amount per area (µg/cm^2^) and the steady-state flux (µg/cm^2^/h) were examined, see Figure 6a,b, respectively. The increase in the cumulative amount per area of all formulations was quite stable until the 8 h point. They did not differ in the early stage at 2 h significantly (*p* > 0.05). The effect of the concentration of solid lipid (N2 and N16) and liquid lipid (N6 and N12) did not considerably influence the cumulative amount per area, including the steady-state flux. However, the amount of surfactant might affect the permeation of TA-loaded NLCs through the mucosa. It could be observed that the low amount of surfactant in the formulation N13 was lower in the cumulative amount per area than the formulation N12 individually at 6 h. The result correlated with the release study in the previous section: a higher amount of surfactant caused an increase in the cumulative release. The result of a higher amount of surfactant agreed with another report that a higher amount of surfactant could increase the permeation by the partitioning effects of the drug between the aqueous and melted lipid phases during the preparation of particles [9]. The effect of a higher amount of surfactant caused the drug to be more soluble, the amount of the drug in the outer layer of the lipid matrix to increase, and for the drug to be released rapidly and easily [9,26,30]. Additionally, mucosa permeation could be increased by using surfactant through the mechanism of inducing the partially reversible gap between cell membranes leading to the increase mucosal absorption of drugs [25,31]. In the early time in 4 h of the permeation study, almost all of the TA-loaded NLCs formulation had higher permeation than the TA in the soybean oil except for the TA-loaded NLCs in the formulation N13. The possible absorption mechanism of this result may have involved the vesicles as drug carriers and absorption enhancers by surfactants [32] since the TA-loaded NLCs formulation was of higher permeation than the TA in soybean oil. However, after 6 h, the TA in soybean oil had significantly higher permeation through the buccal mucosa than the other TA-loaded NLC formulations. Generally, drugs can penetrate through the buccal membrane with passive transport by two major routes, that is, the paracellular (between the cells) and the transcellular (across the cells) routes [5,33]. Among the many factors affecting drug absorption through the cell membranes is lipophilicity. The cell membranes prefer high (log P) value lipophilicity of the drug molecules that pass the cells [5] and this study’s model drug (TA) shows this property. Hence, the TA in the soybean oil can pass the buccal cells thoroughly. Therefore, two possible reasons might describe this occurrence, that is, the lipophilicity of the drug and the limitation of the particles through the cells. The results will be confirmed by using CLSM in a further study.

### 2.6. Confocal Laser Scanning Microscopy (CLSM) Study

**Figure 7:**
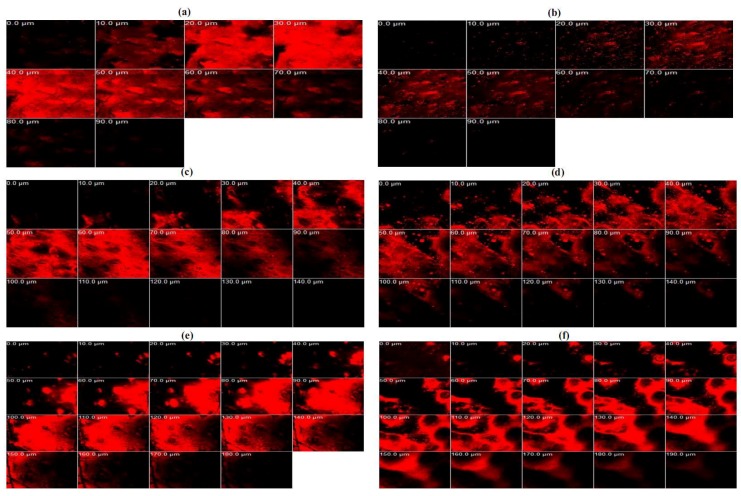
The confocal laser scanning microscopy images of the penetration of porcine buccal mucosa treated with Nile red-loaded NLCs (**a**,**c**,**e**) and Nile red in soybean oil (**b**,**d**,**f**) for 2 h (first row), 4 h (second row) and 8 h (third row).

**Figure 8:**
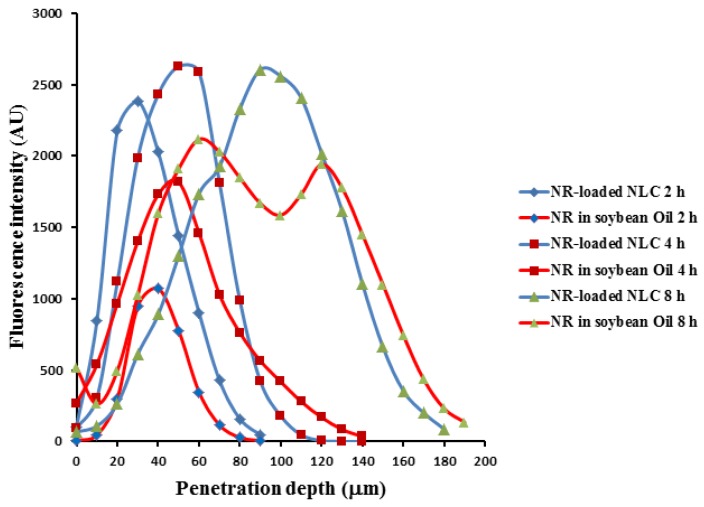
The fluorescence intensity profiles of Nile red at various depths into the buccal mucosa shown in Figure 7.

CLSM was used to observe the drug penetration within porcine buccal mucosa (see Figure 7). TA-loaded NLCs in formulation N2 were selected for study in this experiment compared with TA in soybean oil by changing the active ingredient (TA) in each formulation to Nile red as equal concentration (0.1% *w*/*w*). Before beginning the test, the porcine buccal mucosa without Nile red was used to test the auto-fluorescence response from the mucosa. It could be observed that there was no auto-fluorescence response from the mucosa at the range of excitation and emission wavelength as used (data not shown). The penetration of NR-loaded NLCs in 2 h and 4 h could be observed at the maximum depths of 90 and 140 µm as well as the result of NR in oil. However, the fluorescence intensity of NR-loaded NLCs at the same time was higher than NR in oil in both times, as Figure 8 shows. The result supported the data of the in vitro permeation studies section, that the cumulative amount per area in 4 h of the permeation study of TA-loaded NLCs was higher than the TA in soybean oil. The maximum penetration depths of NR-loaded NLCs and NR in oil in 8 h were 180 and 190 µm, as we see in Figure 8. Nevertheless, the maximum fluorescence intensity of NR-loaded NLCs was also higher than NR in oil at 8 h. After a penetration depth of 120 µm, the fluorescence intensity of NR-loaded NLCs was lower than NR in oil. The result was in line with the previous section of the permeation study: after 6 h, the TA in soybean oil had a higher permeation through buccal mucosa than the TA-loaded NLC formulations. It could be concluded that the rate-limiting steps of NLCs penetration efficiency through porcine buccal mucosa was time and penetration depth. However, other factors may be involved in the penetration rate, such as the lipophilicity of the drug, the size, the net charge, the hydrogen bonding potential, and the structural conformation [5].

## 3. Materials and Methods

### 3.1. Materials

Triamcinolone acetonide was manufactured by the Farmabios Company (Gropello Cairoli PV, Italy) and purchased from PC Drug Co., Ltd. (Bangkok, Thailand). Spermaceti and Tween 80 was purchased from PC Drug Co., Ltd. Soybean oil was obtained from a local market (Bangkok, Thailand). Nile red (NR) was purchased from TCI (Tokyo, Japan). All other chemicals used were of analytical grade and used as received.

### 3.2. Preparation of TA Loaded NLC

A hot homogenization method was used to prepare the NLCs in this study. Briefly, two phases of water and oil were prepared independently. The component of oil phase was spermaceti, soybean oil, and TA (0.1% *w*/*w*), and the water phase was composed of Tween 80 and distilled water. Both phases were heated separately to 80 °C until the temperature of the two phases reached the determined point. The oil phase was added to the water phase and mixed together with a high-shear homogenization at 8000 rpm for 2 min (Ultra-Turrax^®^ T25, IKA-Works, Inc., Staufen im Breisgau, Germany). The size of the pre-emulsion was reduced by using a probe sonicator (Ultrasonic Processor 400, Hielscher, Teltow, Germany) at 100% amplitude for 15 min. Then, the NLC dispersion was cooled down at room temperature before the next experiment. The TA dissolved in soybean oil (as did an equal concentration of TA) was prepared to be compared with the TA-loaded NLCs in the section of the in vitro release and permeation study.

### 3.3. Experimental Design and Statistical Analysis

Various factors like the concentration of solid lipid, oil, and emulsifier were identified as critical to give a product in the nano range. So, three main factors affecting particle size, zeta potential, and entrapment efficiency were selected to use for the optimization of the TA-loaded NLCs formulation. Based on the number of factors and their level, a Box–Behnken design was used to evaluate the effect of the formulation affecting the physical properties of the TA-loaded NLCs. Table 3 shows the three main independent factors of the concentration of spermaceti (X_1_), soybean oil (X_2_), and Tween 80 (X_3_) that were operated at the three levels (+1, 0 and −1). The particle size (Y_1_), zeta potential (Y_2_), and % entrapment efficiency (Y_3_) were used as the dependent responses. The responses of all model formulations were treated by Design-Expert^®^ software (version 9; Stat-Ease, Inc., Minneapolis, MN, USA). A total of 17-run, 3-level and 3-factor with 5 center points were created by the program and the tests were run in random order. Table 1 displays the experimental design and response data.

### 3.4. Physicochemical Characterization of TA Loaded NLC (Zeta Potential and Particle Size)

The TA-loaded NLCs were characterized with respect to their particle size using the light scattering technique (LA-950, Horiba, Ltd., Kyoto, Japan) and they were dispersed in distilled water with gentle stirring before the test. The zeta potential was measured by the Zeta Plus (Brookhaven Instruments Co, Holtsville, NY, USA) and the TA-loaded NLCs were dispersed in distilled water with gentle stirring at a volume ratio of 1:50 before measurement. All measurements are carried out in triplicate (*n* = 3).

### 3.5. Determination of Entrapment Efficiency

The amount of TA in NLCs was determined by adding methanol in the NLC dispersion for destroying the structure of NLCs. Briefly, 1 mL of the NLC dispersion was mixed with 4 mL of methanol by using the vortex mixer for 2 min. The released TA was centrifuged at 14,000 rpm for 15 min (Model 6000, KUBOTA, Tokyo, Japan) to separate it from the NLCs. The supernatant of the released TA was decanted and quantified by UV spectrophotometry at 254 nm (UV-1800, Shimadzu, Kyoto, Japan). The percentage of the entrapment efficiency (%EE) was calculated using the following equation:

(1) $$\mathrm{EE}\;(\%)=\frac{\mathrm{Released}\;\mathrm{TA}\;}{\mathrm{Total}\;\mathrm{amount}\;\mathrm{of}\;\mathrm{TA}}\times 100$$

### 3.6. Field Emission Scanning Electron Microscope (FESEM) and Energy-Dispersive X-ray Spectroscopy (EDS)

The FESEM (model JSM 7800F, JEOL, Tokyo, Japan) equipped with EDS (X-Max 20, Oxford Instruments, Abingdon, UK) was used to examine the morphological characteristic and the identified TA of the selected TA-loaded NLCs (N2). The microscope performed with an accelerating voltage of 2 keV. The samples were dropped on a metal stub with double-sided adhesive tape and dried at an ambient temperature until the water completely evaporated from the sample. Then, the dried sample was coated with a fine gold layer under the vacuum before obtaining the micrographs.

### 3.7. In Vitro Release of TA-Loaded NLCs

A modified Franz-type diffusion cell was used to measure the release of TA from the NLC dispersion. The dialysis membrane (Cellu-Sep^®^ T2, MWCO 6–8 kDa, Membrane Filtration Products Inc., Seguin, TX, USA) was cut into a 1.5 cm diameter circle and placed between the donor and receptor chamber. The receiver chamber was filled with 15 mL of 50% *v*/*v* ethanol in simulated saliva fluid (SSF) pH 6.80. A water jacket was used to maintain the diffusion cell at 37 °C and stirred with a magnetic stirrer in the receptor chamber. The selected TA-loaded NLCs (N2, N6, N12, N13, and N16) and TA in soybean oil (1.5 mL) were placed in a donor chamber. Half of one milliliter of the release medium was removed and replaced immediately by equal volumes of fresh SSF at predetermined periods of time. The amount of TA was analyzed spectrophotometrically at 254 nm (Shimadzu UV-1800). The release kinetics of the selected NLC formulations were analyzed to fit with a linear regression equation of zero-order (Equations (2)), first-order (Equations (3)), or Higuchi diffusion models (Equations (4) and (3)). The suitable mathematical release model was chosen from a maximum R^2^ of that linear regression equation:

Q = Q_0_ + K_0_t (2)

Log Q = log Q_0_ − K_1_t/2.303 (3)

Q = K_h_t^1/2^ (4)

where Q is cumulative amount of drug release at time (t), t = time in hours, Q_0_ = initial amount of drug, K_0_ = zero order release constant, K_1_ = first order release constant, and K_h_ = Higuchi constant.

### 3.8. In Vitro Permeation Studies

A Franz-type diffusion cell method was also used to investigate in vitro permeation studies of the selected TA-loaded NLC formulations (N2, N6, N12, N13, and N16) and TA in soybean oil. This method was applied from our study, as previously reported [2]. Porcine buccal mucosa, obtained from a local slaughterhouse (Nakhon Pathom, Thailand), was used as permeability barrier biological membrane due to likeness with human buccal tissue [2,12]. The buccal mucosae were sectioned and washed with 0.9% *w*/*v* NaCl to remove the underlying connective tissues for isolating the mucosal membrane. The thickness of the membrane used is approximately 500–600 µm. The isolated mucosal membrane was placed in normal saline solution at 4 °C before use in permeation experiments. The receiver chamber was filled with 15 mL of 50% *v*/*v* ethanol in the simulated saliva fluid (SSF) pH 6.80. A water jacket was used to maintain the diffusion cell at 37 °C and stirred with a magnetic stirrer in the receptor chamber. The isolated porcine buccal mucosa was mounted between the donor and the receptor chamber. The selected TA-loaded NLCs (1.5 mL) and TA in soybean oil were placed in a donor chamber. Half of one milliliter of the release medium was removed and replaced immediately by equal volumes of fresh SSF at predetermined periods of time. The amount of TA was analyzed spectrophotometrically at 254 nm (Shimadzu UV-1800). The results of the permeation are displayed as the cumulative amount per area (mg/cm^2^) versus time (minute) and as the steady state flux (mg/cm^2^/h).

### 3.9. Confocal Laser Scanning Microscopy (CLSM) Study

CLSM is a useful technique for the visual examination of the fluorescence dyes’ penetration through the skin or buccal mucosa because they have many advantages, such as the facility of sample preparation, non-invasiveness, high resolution of the image, and the visualization of images in different depths without mechanical dividing [12]. Nile red (NR) was employed as a fluorescent dye in this study since it is a popular fluorescent agent used as a model of hydrophobic substance [12,32]. From the result of the release study, the selected TA-loaded NLCs formulation (N2) was selected for study in this experiment compared with the TA dissolved in soybean oil by changing the active ingredient (TA) in each formulation to NR as equal concentration (0.1% *w*/*w*).

In this study, the penetration of the Nile red was investigated as described in the section on in vitro permeation studies. After treating the NR in soybean oil and NR-loaded NLCs on the buccal membrane, at 2 h, 4 h, and 8 h, the dye was carefully removed, washed twice with SSF, and the mucosal surface cleaned with a cotton bud. Then, the treated buccal membrane was removed from the Franz-type diffusion cell and kept in lower −20 °C before investigation using CLSM.

The penetration study using CLSM was applied from T. Subongkot, as previously reported in [32]. Briefly, a piece of the treated buccal mucosa was placed on a coverslip. CLSM (Carl Zeiss, Jena, Germany) with a Helium-Neon laser at an excitation wavelength of 543 nm and an emission wavelength of 580 nm equipped with an inverted microscope using the 10× objective lens to scan the membrane. To compare the distribution, depth of penetration, and fluorescence intensity, the laser was used to scan through the membrane to provide a series of x-z plane serial images of the NR. With respect to the intensity of the fluorescence, the horizontal line of each image using Zeiss LSM 5 operating software determined the fluorescence intensity and then each value was plotted as a function of the penetration depth.

### 3.10. Statistical Analysis

The data were expressed as the mean ± S.D. of the three determinations (*n* = 3). An analysis of the variance (ANOVA) was used to determine the statistical analysis at the 0.05 significant levels.

## 4. Conclusions

The TA-loaded NLCs for the buccal drug delivery system were prepared by the hot homogenization method. Box–Behnken design was used to evaluate the effect of the formulation affecting the physical properties of TA-loaded NLCs. The particle size of the TA-loaded NLCs was displayed in the size range of 80.5–185.8 nm. The particle size was smaller when the concentration of Tween 80 was increased. The result correlated with the result of increasing the amount of liquid lipid; the particle size was smaller when it was higher. The zeta potential ranged between −5.91 and −20.83 mV and the %EE of TA-loaded NLCs were in the range of 82.52–101.09%, indicating that the NLC system had a highly effective encapsulation of TA. The FESEM confirmed that the sizes of the TA-loaded NLCs were around 100 nm and EDS confirmed that TA could be incorporated in the NLC system. The Higuchi model gave the highest value of the R^2^, indicating that this model was a fit for the TA release profiles of TA-loaded NLCs. During the early time (4 h) of the permeation study, almost all the TA-loaded NLC formulations had a higher permeation than the TA in soybean oil. The penetration of the NR-loaded NLCs at 2 h and 4 h could be observed at the maximum depths of 90 and 140 µm, as well as in the result for NR in the oil. However, the fluorescence intensity of the NR-loaded NLCs at the same time was higher than the NR in the oil at both times. In conclusion, the TA-loaded NLCs is a promising efficiency approach for the buccal drug delivery system.

## Figures and Tables

**Table 1:** The Box–Behnken experimental design and response data.

Formulation	Actual Value of Independent Factors			Response Values		
	Spermaceti (g), X_1_	Soybean Oil (g), X_2_	Tween 80 (g), X_3_	Particle Size (nm), Y_1_	Zeta Potential (mV), Y_2_	EE (%), Y_3_
N1	1.0	0.25	3.0	80.75	−17.91	86.89
N2	1.0	0.50	4.0	81.15	−17.98	93.96
N3	1.0	0.50	2.0	93.35	−14.49	90.77
N4	1.0	0.75	3.0	91.60	−8.16	93.07
N5	1.5	0.25	2.0	185.20	−20.83	90.94
N6	1.5	0.25	4.0	80.50	−17.07	98.76
N7	1.5	0.50	3.0	95.10	−6.90	84.45
N8	1.5	0.50	3.0	82.70	−7.09	97.84
N9	1.5	0.50	3.0	80.95	−15.7	88.95
N10	1.5	0.50	3.0	86.95	−15.18	82.52
N11	1.5	0.50	3.0	81.85	−17.77	88.72
N12	1.5	0.75	4.0	82.15	−5.91	101.09
N13	1.5	0.75	2.0	83.15	−17.67	91.61
N14	2.0	0.25	3.0	184.75	−18.47	98.40
N15	2.0	0.50	2.0	185.80	−17.83	98.36
N16	2.0	0.50	4.0	82.25	−12.60	90.10
N17	2.0	0.75	3.0	82.55	−16.64	95.14

**Table 2:** The in vitro release kinetics of triamcinolone acetonide-loaded nanostructured lipid carriers.

Mathematical Release Models	N6 (R^2^)	N12 (R^2^)	N16 (R^2^)	N13 (R^2^)	N2 (R^2^)
Zero-order	0.9351	0.942	0.9715	0.9736	0.9522
First-order	0.8572	0.8153	0.9547	0.9481	0.8073
Higuchi	0.9823	0.9561	0.9812	0.967	0.9329

**Table 3:** The experimental variables and their levels.

Independent Factors	Levels		
	−1	0	+1
Spermaceti (g), X_1_	1.0	1.5	2.0
Soybean oil (g), X_2_	0.25	0.50	0.75
Tween 80 (g), X_3_	2.0	3.0	4.0
